# GLADS: A gel-less approach for detection of STMS markers in wheat and rice

**DOI:** 10.1371/journal.pone.0224572

**Published:** 2019-11-05

**Authors:** Gautam Vishwakarma, Ravi Prakash Sanyal, Ajay Saini, Parmeshwar Kumar Sahu, Ravi Raj Singh Patel, Deepak Sharma, Ratan Tiwari, Bikram Kishore Das

**Affiliations:** 1 Nuclear Agriculture and Biotechnology Division, Bhabha Atomic Research Centre, Trombay, Mumbai, Maharashtra, India; 2 Homi Bhabha National Institute, Anushaktinagar, Trombay, Mumbai, Maharashtra, India; 3 Molecular Biology Division, Bhabha Atomic Research Centre, Trombay, Mumbai, Maharashtra, India; 4 Department of Genetics and Plant Breeding, Indira Gandhi Krishi Vishwavidyalaya, Raipur, Chhattisgarh, India; 5 ICAR - Indian Institute of Wheat and Barley Research, Karnal, Haryana, India; Louisiana State University, UNITED STATES

## Abstract

Sequence tagged microsatellite site (STMS) are useful PCR based DNA markers. Wide genome coverage, high polymorphic index and co-dominant nature make STMS a preferred choice for marker assisted selection (MAS), genetic diversity analysis, linkage mapping, seed genetic purity analysis etc. Routine STMS analysis involving low-throughput, laborious and time-consuming polyacrylamide/agarose gels often limit their full utility in crop breeding experiments that involve large populations. Therefore, convenient, gel-less marker detection methods are highly desirable for STMS markers. The present study demonstrated the utility of SYBR Green dye based melt-profiling as a simple and convenient gel-less approach for detection of STMS markers (referred to as GLADS) in bread wheat and rice. The method involves use of SYBR Green dye during PCR amplification (or post-PCR) of STMS markers followed by generation of a melt-profile using controlled temperature ramp rate. The STMS amplicons yielded characteristic melt-profiles with differences in melting temperature (Tm) and profile shape. These characteristic features enabled melt-profile based detection and differentiation of STMS markers/alleles in a gel-less manner. The melt-profile approach allowed assessment of the specificity of the PCR assay unlike the end-point signal detection assays. The method also allowed multiplexing of two STMS markers with non-overlapping melt-profiles. In principle, the approach can be effectively used in any crop for STMS marker analysis. This SYBR Green melt-profiling based GLADS approach offers a convenient, low-cost (20–51%) and time-saving alternative for STMS marker detection that can reduce dependence on gel-based detection, and exposure to toxic chemicals.

## Introduction

Conventional breeding utilizes various approaches for improvement of desired traits in crop plants [1,2], which involve laborious and time-consuming manual screening of large experimental populations. In addition, these are not straight forward methods for introgression of complex traits/phenotypes governed by multiple genes [3]. DNA markers ascertains linked traits (at any stage of plant) saves screening time, and hence are highly desirable for crop breeding applications such as, marker assisted selection (MAS) or marker assisted backcross breeding (MABB) [4]. Polymerase chain reaction (PCR) has revolutionized the development and use of a variety of DNA markers viz. Sequence Tagged Microsatellite Site (STMS), Sequence-Tagged Site (STS), Sequence Characterized Amplified Region (SCAR), Randomly Amplified Polymorphic DNA (RAPD), Arbitrary Primed-PCR (AP-PCR), Inter Simple Sequence Repeat (ISSR), Amplified Fragment Length Polymorphism (AFLP), Retroposon-Microsatellite Amplified Polymorphism (RE-MAP) [5]. Recent advances in genome sequencing has resulted in development of SNP markers for diverse applications including genome wide association studies (GWAS), marker development, genomic selection, germplasm characterization [6,7].

STMS markers due to their co-dominant nature, high polymorphism, high abundance, and dispersed genomic distribution [8] are highly popular, and have been extensively used for high density maps, tagging genes, map based cloning, gene pyramiding, species identification [9–10] etc. For applications like MAS of a trait or MABB, STMS marker are more preferred owing to their convenience in use and cost effectiveness [9,10–12]. Furthermore, STMS also exhibit ‘transferability’, a useful attribute that allow the markers developed in one species to be utilized in related species [13,14]. For example, STMS markers from barley exhibit considerable transferability in wheat (~78%), rye (~75%) and rice (~42%) [15,16]. Despite advantages, STMS markers have not been utilized to their full potential, partly due to the non-availability of sequence of flanking regions (for locus specific primer designing) in several non-sequenced genomes, and to a certain extent due to the complexities associated with gel-based detection methods [17–19]. Availability of next generation sequencing (NGS) approaches have resulted in development of large number of STMS or SSR markers (genomic/EST derived) in several non-model crops having limited genomic information. These markers are important genetic resources for linkage mapping, genetic diversity, MAS, DNA fingerprinting, and other applications for these crops [13,20,21].

PCR amplified amplicons (single- and multi-locus markers) are routinely scored on agarose or polyacrylamide gels [17–19]. The gel-based methods are low-throughput, laborious, and add additional cost, thereby limiting the utility of even single-band markers to their full potential, as needed in applications such as MAS [22]. Scoring STMS markers on long-format polyacrylamide/high-resolution agarose gels is cumbersome, time-consuming and not suitable for high-throughput analysis [23]. Several gel-less approaches have been reported for DNA marker detection [19 and references therein], however majority of them are technically intensive. Hernández and co-workers reported a simplified assay involving addition of ethidium bromide to detect amplified products directly in the PCR tubes [24]. Advent of real-time PCR and sensitive fluorescent dyes [25] resulted in development of a diverse array of DNA detection methods based on hybridization probes, molecular beacons, TaqMan probes, scorpion probes, SYBR Green and High-resolution melting or HRM [26]. Capillary electrophoresis and use of fluorescently labelled primers were shown to automate the STMS marker scoring to a certain extent [27–29]. Although, technical feasibility and utility of these approaches in STMS analysis have been demonstrated [30–33] these are technologically intensive, expensive, require specialized instrumentation, and not suitable for analysis of large breeding populations [31,34,35]. In view of the above-mentioned concerns of different methods, simple, convenient and low cost gel-less detection methods are desirable for STMS scoring. These will be important for effective utilization of STMS in MAS programs that handles large experimental populations.

SYBR Green, a low-cost DNA binding dye, is an ideal candidate for large scale analysis [36]. Previously, we showed the utility of SYBR Green based melt-profiling as an effective method for SCAR marker detection in a gel-less manner, [19]. The approach is based on existing primers and is cost-effective than other methods [19]. The present study explores the utility of SYBR Green dye based melt-profiling as a gel-less approach for detection of STMS markers (referred to as GLADS). This approach involves generation of specific melt-profiles of wheat and rice STMS markers for their detection. The STMS markers/alleles were differentiated based on the melting temperature (Tm) and profile characteristics. The approach also demonstrated the feasibility of multiplexing that can further reduce the cost and time of analysis. Overall, SYBR green melt-profiling based GLADS can serve as a convenient gel-less method for detection of STMS markers linked to desirable traits. It is technologically less intensive, saves time, cost-effective, and therefore suitable for rapid analysis of large number of experimental samples as in MAS based crop breeding experiments.

## Materials and methods

### Plant material

A total of twelve bread wheat (*Triticum aestivum* L.) and twelve rice (*Oryza sativa* L.) genotypes were used in the present study. Seeds of the genotypes were obtained from the following Institutes: 1) Indian Agricultural Research Institute (IARI), New Delhi, India, 2) ICAR-IARI Regional Station, Wellington, Tamil Nadu, India, 3) Punjab Agricultural University (PAU), Ludhiana, India, 4) ICAR-Indian Institute of Wheat & Barley Research (IIWBR) Regional Station, Flowerdale, Shimla, Himachal Pradesh, India, and 5) Indira Gandhi Krishi Vishwavidyalaya (IGKV), Raipur, Chhattisgarh, India (Table 1). The wheat and rice plants were grown at experimental field at Bhabha Atomic Research Centre (BARC), Mumbai, Maharashtra.

**Table 1:** List of wheat (W1 –W12) and rice (R1 –R21) genotypes used for analysis.

S. No.	Genotypes	Source
W1	Kalyansona-1 (KS-1)	ICAR-IARI, New Delhi, India
W2	Sonalika	
W3	C-306	
W4	TWS	BARC, Trombay, Mumbai, India
W5	Vaishali	ICAR-IARI, New Delhi, India
W6	Kite	ICAR-IIWBR Regional Station, Shimla, Himachal Pradesh, India
W7	Flinder	
W8	HW-2021	ICAR-IARI Regional station Wellington, Tamil Nadu, India
W9	NIAW-917	
W10	PBW-343	PAU, Ludhiana, India
W11	Chinese Spring	ICAR IARI Regional station Wellington, Tamil Nadu, India
W12	LWH	
R1	Jonyaphool	IGKV, Raipur, Chhattisgarh, India
R2	Tulsimongra	
R3	Sihar	
R4	Bhusu	
R5	Badshahbhog-2	
R6	Bhajna	
R7	Pangudi Goindi	
R8	Khetganga	
R9	Baigani Dhan	
R10	Jhunuprash	
R11	Karhani	
R12	Alsenga	

Note: ICAR: Indian Council of Agricultural Research, IARI: Indian Agricultural Research Institute, PAU: Punjab Agricultural University, IIWR: Indian Institute of Wheat and Barley Research, BARC: Bhabha Atomic Research Centre, IGKV: Indira Gandhi Krishi Vishwavidyalaya. TWS: Trombay Wheat Selection, LWH: Local Wheat Hango.

### DNA isolation and quantification

Total genomic DNA was isolated from one-month old seedlings grown in experimental field at BARC as per protocol detailed in Eswaran and co workers [37], with minor modifications. Briefly, 200 mg leaf tissue was homogenized in 2.0 ml DNA extraction buffer (100 mM Tris, 20 mM EDTA, 0.5 M NaCl, 7 M Urea, 0.1% β-mercaptoethanol and 2% SDS), subjected to phenol:chloroform:isoamyl alcohol extraction, and followed by precipitation of genomic DNA by addition of sodium acetate (3 M, 0.1 volume) and isopropanol (0.7 volume). The DNA was recovered by centrifugation, dissolved in TE buffer (Tris-Cl:10 mM, EDTA: 1 mM, pH: 8.0) and treated with 50 μg of RNase (Roche Diagnostics, Mannheim, Germany) to remove the residual RNA contamination. DNA preparation was assessed for quantity (A_260_ nm) and quality (A_260_ nm/A_280_ nm) on a spectrophotometer (UV-1800, Shimadzu, Tokyo, Japan), and the integrity was assessed by electrophoresis on a 0.8% agarose gel (Sigma-Aldrich, St. Louis, MO, USA). DNA samples with good quality and integrity were used for analysis.

### Oligonucleotide primers for wheat and rice STMS loci

Oligonucleotide primer pairs for PCR amplification of STMS markers were synthesized from Bangalore Genei Pvt. Ltd. (Bengaluru, India). Oligonucleotide primer-pairs for fifty-five wheat and eighteen rice STMS marker loci were synthesized as per the sequences given in previous publications [38,39]. General characteristics of wheat and rice STMS loci analyzed are listed in S1 Table.

### Polymerase chain reaction: Optimization of amplification and multiplexing

PCR amplification of STMS markers was performed on Mastercycler gradient PCR machine (Eppendorf, Hamburg, Germany) using reaction components from Bangalore Genei Pvt. Ltd. (Bengaluru, India). The PCR reaction mix (volume: 25 μl) contained genomic DNA (50–75 ng), dNTPs (250 μM each), 10X reaction buffer (15 mM Tris-Cl pH 9.0, 50 mM KCl, 0.01% gelatin, 2.0–3.0 mM MgCl_2_), 3–5 pmol of each primer and 1.0 unit of *Taq* DNA polymerase. Following thermal cycling conditions were used for PCR amplification: initial denaturation at 94 °C (5 min), 40 cycles of denaturation (94 °C, 30 sec), annealing (62 °C, 45 sec), extension (72 °C, 40 sec), and final extension at 72 °C (7 min). Annealing temperature for PCR amplification was optimized by gradient PCR. Multiplex PCR analysis was attempted for different combinations of STMS markers using the same conditions described above.

### Agarose gel electrophoresis

The amplification of PCR products was verified by electrophoresis at 8–10 V cm^-1^ in 1X TBE buffer, on 2.0% agarose gel (Sigma-Aldrich, St. Louis, MO, USA). The DNA fragments were stained with ethidium bromide and, photographed under UV light on a gel-documentation system from Syngene (Syngene, Cambridge, UK), and the sizes were estimated by GeneTools software (Syngene, Cambridge, UK).

### SYBR Green dye-based melt-profiling of microsatellite amplicons

For in-tube and gel-less detection of STMS markers, SYBR Green (S9430, Sigma-Aldrich, St. Louis, MO, USA), a non-specific DNA binding fluorescent dye, was included in the PCR reaction mix (described above) at 1X final concentration. PCR assays were carried out on Mastercycler *ep* Realplex^4^ (Eppendorf, Hamburg, Germany) and LightCycler 480 II (Roche, Mannheim, Germany) PCR instruments, as detailed above. After the PCR, melt-profile analysis was carried out by measuring the fluorescence intensity of the amplicons in continuous mode (temperature range: 60 °C to 95 °C). Different temperature ramp rates (1.75 °C min^-1^, 3.5 °C min^-1^ and 7.0 °C min^-1^) were used to optimize the melt-profile analysis. The raw melt-profile data was transformed into the negative first derivative data (as a function of temperature) to identify maximum intensity changes (represented as peak curves) using the Eppendorf Mastercycler *ep* realplex software (version 2.2) or LightCycler^®^ 480 Software (version 1.5) for assays carried out on LC 480 II instrument. For uniformity in data presentation, the fluorescence intensity was plotted as percent values versus temperature. Melt-profile analysis was also performed for STMS amplicons amplified by multiplex PCR assay, by using the optimized temperature ramp conditions as described above. In an alternative strategy, the PCR amplification of STMS markers was carried using normal PCR assay, SYBR Green dye was added post-PCR followed by melt-profiling on a real-time instrument as mentioned above. The STMS melt-profiling experiments were carried out using two independent DNA preparations and repeated at least two times, and appropriate controls were included for each set of analysis.

## Results

### Optimization of conditions for PCR amplification of STMS markers

**Fig 1:**
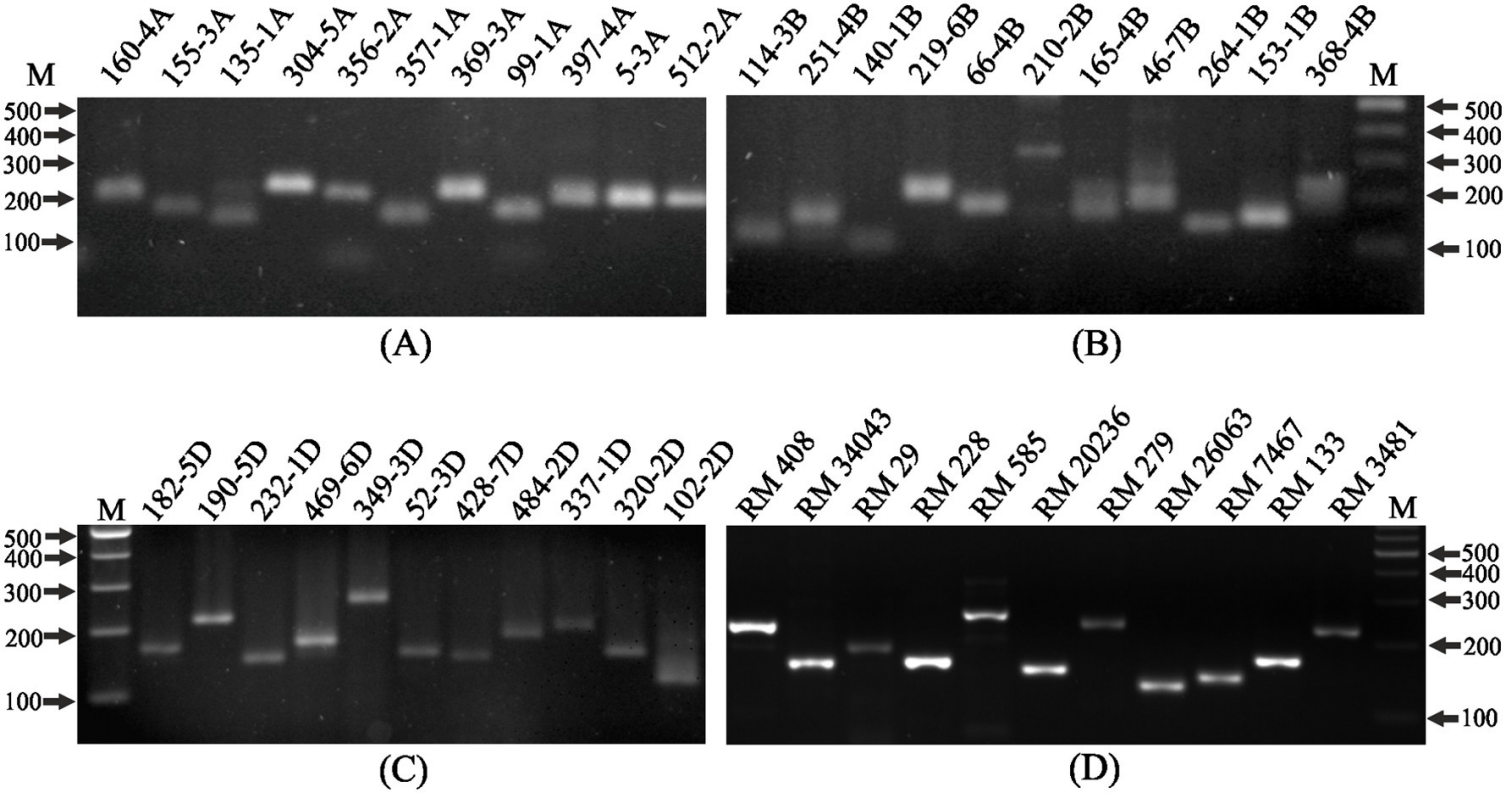
Analysis of PCR amplified STMS markers on 2% agarose gel. A) wheat A-genome STMS markers, B) wheat B-genome STMS markers, C) wheat D-genome STMS markers, and D) rice STMS markers. Designations of the STMS markers are indicated on the top of the lanes. Lane ‘M’ indicates 100 bp ladder.

Optimization of PCR conditions is necessary to minimize non-specific amplification products and primer-dimers, particularly when sensitive dyes such as SYBR Green are used in the assay. Using representative wheat and rice genotypes, amount of genomic DNA, primer concentration and annealing temperature were optimized for STMS PCR amplification. Use of 50–75 ng genomic DNA and 3–5 pmol primer resulted in optimum amplification with minimum non-specific signal. Gradient PCR assay (annealing temperature range: 58°C to 62°C) was carried out to find optimum annealing temperature for amplification. Annealing temperature of 62°C enhanced specificity and minimized non-specific products in PCR amplification of most STMS (S1 Fig). Using optimized conditions, the STMS markers specific to A-, B- and D-genomes of wheat yielded clean profiles on PCR amplification from a representative genotype (Fig 1A, 1B and 1C). Under optimized PCR conditions the rice STMS markers also yielded clean PCR amplification profiles (Fig 1D).

SYBR Green dye at 1X concentration was optimum (good fluorescence signal and negligible effect on PCR) for STMS melt-profile analysis. The melt-curve analysis was carried out using different ramping rates (1.75°C min^-1^, 3.5°C min^-1^ and 7.0°C min^-1^). Ramp rate of 3.5°C min^-1^ was found to be optimal for melt-profiling as it yielded low background signal and few non-specific signal spikes. The melt-profiling approach also showed enhanced specificity at higher annealing temperature in PCR, as shown for two wheat STMS markers (S2A and S2B Fig). However, certain STMS markers that showed a doublet on agarose gel yielded two peaks on SYBR Green melt-profile analysis despite increase in annealing temperature (S2C Fig).

### SYBR Green melt-profiling yielded STMS marker specific profiles

**Fig 2:**
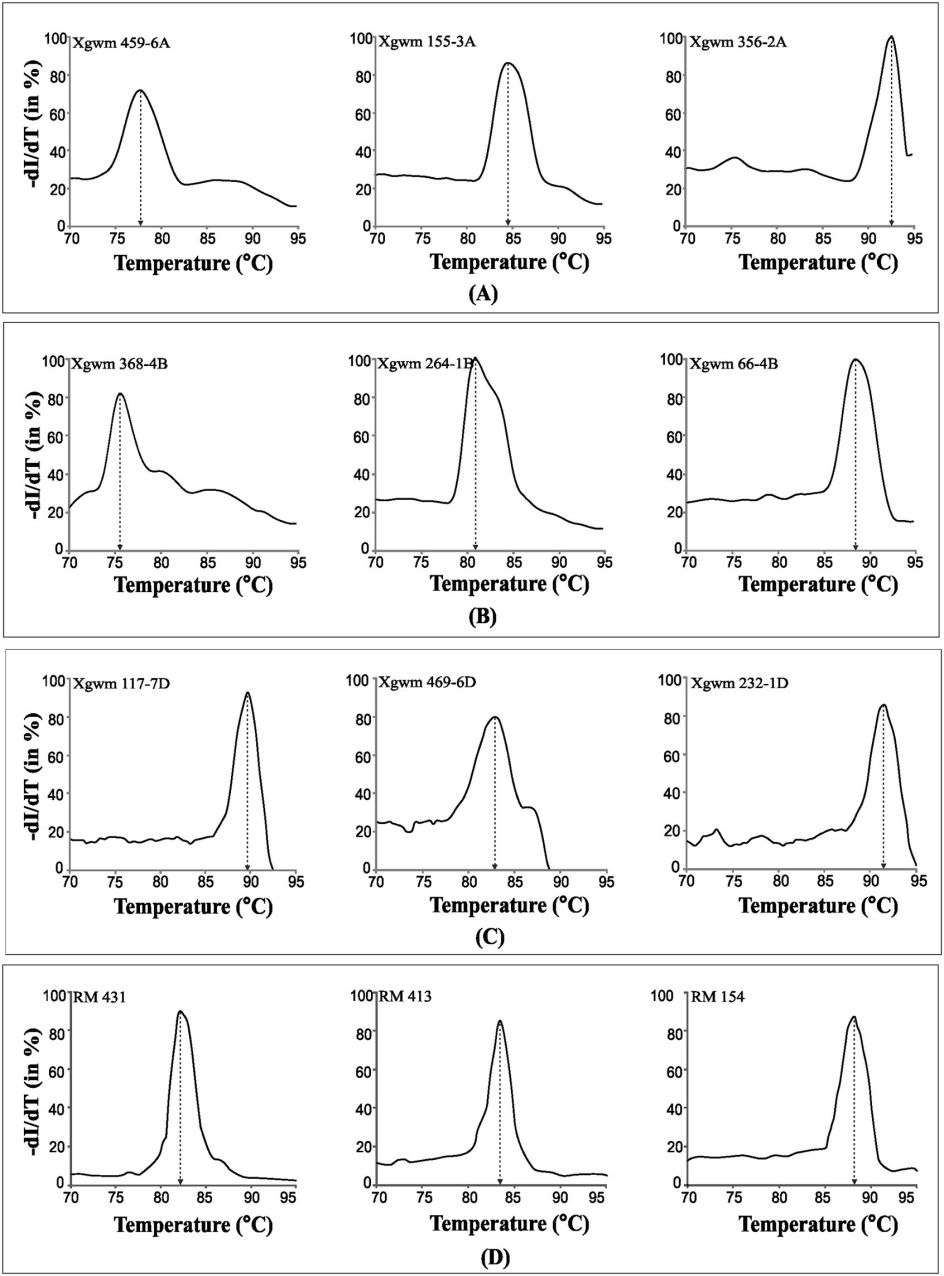
SYBR Green melt-profiles specific to different STMS markers. A) melt-profiles of three STMS markers specific to wheat A-genome, B) melt-profiles of three STMS markers specific to wheat B-genome, C) melt-profiles of three STMS markers specific to wheat D-genome, and D) melt-profiles of three rice STMS markers. The STMS markers were amplified using a representative genotype. Designation of STMS is indicated on upper left corner of each plot and the Tm is indicated by vertical dashed line.

The STMS markers that yielded clean amplification on agarose gel were included for SYBR Green melt-profiling analysis. SYBR Green melting curve analysis was carried for thirty-seven STMS marker loci from A-, B- and D-genomes of wheat, and for seven STMS loci from rice (Table 2), as detailed in materials and methods. The SYBR Green melt-profiling approach yielded characteristic profiles of STMS markers of wheat and rice. As an example, Fig 2A–2C show melt-profiles of three STMS markers specific to A-genome, B-genome, D-genome of wheat, and Fig 2D shows melt-profiles of three rice STMS markers. In general, the melting temperature (Tm) of the STMS profiles ranged from ~75 °C to ~95 °C (Fig 2).

**Table 2:** List of wheat A, B, and D-genome, and rice STMS marker loci analyzed in the present study.

S. No.	STMS marker	Species/Genome	Repeat Motif	No of Alleles	*Amplicon size range (bp)
1	Xgwm 136–1A	Wheat A-Genome	(CT)58	04	320–400
2	Xgwm 357–1A		(GA)18	03	120–140
3	Xgwm 135–1A		(GA)20	05	96–180
4	Xgwm 99–1A		(CA)21	05	96–156
5	Xgwm 265–2A		(GT)23	04	174–250
6	Xgwm 448–2A		(GA)29	03	220–252
7	Xgwm 155–3A		(CT)19	04	108–140
8	Xgwm 162–3A		(CA)14AA(CA)4	04	Null, 220–240
9	Xgwm 160–4A		(GA)21	03	170–230
10	Xgwm 156–5A		(GT)14	03	280–330
11	Xgwm 304–5A		(CT)22	05	180–232
12	Xgwm 459–6A		(GA)>28	03	110–130
13	Xgwm 570–6A		(CT)14(GT)18	03	100–140
14	Xgwm 169–6A		(GA)23	05	Null, 200–250
15	Xgwm 282–7A		(GA)38	05	Null, 190–267
16	Xgwm 140–1B	Wheat B-Genome	(CT)42	04	205–232
17	Xgwm 264–1B		(CA)9A(CA)24	03	Null, 145–210
18	Xgwm 257–2B		(GT)30	02	176–182
19	Xgwm 114–3B		(GA)53	05	Null, 103–147
20	Xgwm 547–3B		(CA)12	02	167–171
21	Xgwm538–4B		(GT)6(T)(GT)10	02	139–180
22	Xgwm 368–4B		(AT)25	05	Null, 202–242
23	Xgwm 68–5B		(GA)3(G)3(GA)25	01	116
24	Xgwm 46–7B		(GA)2GC(GA)33	05	120–150
25	Xgwm 337–1D	Wheat D-Genome	(CT)5(CACT)6(CA)43	03	Null, 200–210
26	Xgwm 232–1D		(GA)19	03	Null, 150
27	Xgwm 642–1D		(GT)14	02	200
28	Xgwm 320–4D		(GT)9(GA)15	03	Null, 175–275
29	Xgwm 645–3D		(CT)23imp	03	Null, 160–150
30	Xgwm 52–3D		(GT)4AT(GT)20	03	Null, 150–160
31	Xgwm 608–4D		(GA)16	02	Null, 175
32	Xgwm 182–5D		(CT)18	02	Null, 175
33	Xgwm 174–5D		(CT)22	03	Null, 400–450
34	Xgwm 192–5D		(CT)46	02	Null, 150
35	Xgwm 190–5D		(CT)22	02	Null, 225
36	Xgwm 55–6D		(TC)3(T)3(CT)17	01	100
37	Xgwm 111–7D		(CT)32(GT)17	03	Null, 100–120
38	RM 55	Rice	(GA)17	04	Null, 170–190
39	RM 154		(GA)21	04	180–190
40	RM 413		(AG)11	03	70–90
41	RM 431		(AG)16	02	250–260
42	RM 259		(CT)17	03	160–190
43	RM 447		(CTT)8	03	100–140
44	RM 514		AC(12)	02	240–260

*Sizes of STMS amplicons were estimated by ‘GeneTools’ software (Syngene, UK)

Results show that the melt-profiles can be useful to differentiate several STMS markers from one another. Several pairs of wheat STMS markers could be differentiated based on the melt-profiles such as: Xgwm 459–6A (77.7 °C) and Xgwm 160–4A (83 °C), Xgwm 459–6A (77.7 °C) and Xgwm 304–5A (90.5 °C), Xgwm 459–6A (77.7 °C) and Xgwm 155–3A (84.5 °C), Xgwm 459–6A (77.7 °C) and Xgwm 135–1A (87.5 °C), Xgwm 459–6A (77.7 °C) and Xgwm 356–2A (92.5 °C), Xgwm 264–1B (80.8 °C) and Xgwm 165–4B (88.5 °C), Xgwm 368–4B (75.5 °C) and Xgwm 66–4B (88.5 °C), Xgwm 368–1B (75.5 °C) and Xgwm 210–2B (85.5 °C), Xgwm 320–2D (82.5 °C) and Xgwm 111–7D (89.5 °C), Xgwm 320–2D (82.5 °C) and Xgwm 174–5D (90.5 °C), Xgwm 320–2D (82.5 °C) and Xgwm 232–1D (91.5 °C), Xgwm 469–6D (82.8 °C) and Xgwm 232–1D (91.5 °C) (Fig 2). These results show that the SYBR Green melt-profiles can be used for gel-less detection of STMS markers, and based on their specific Tm values and melt-curve characteristics many of these can be easily distinguished from one another.

### SYBR Green melt-profiling for detecting STMS allele polymorphism

**Fig 3:**
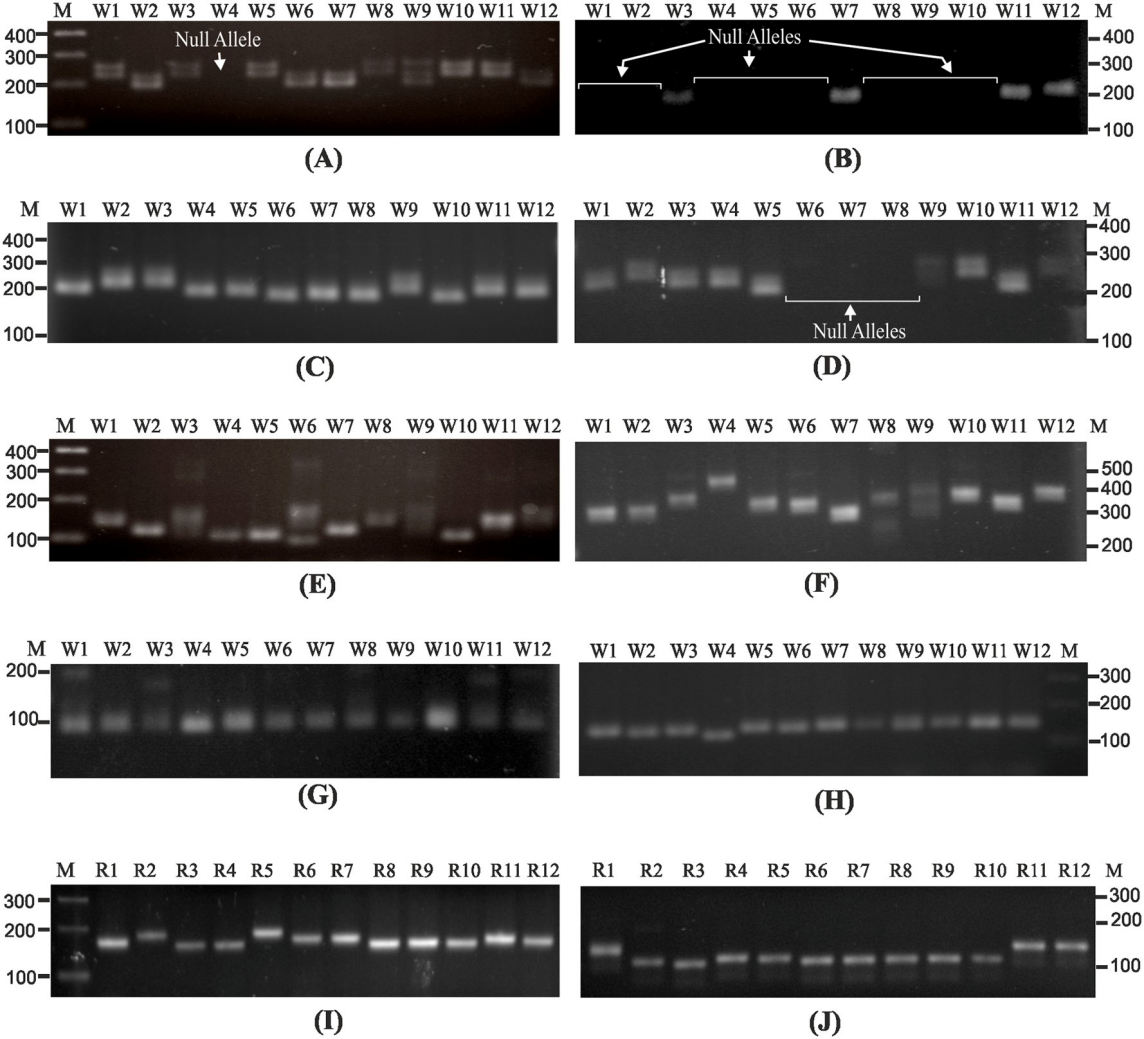
Analysis of allelic polymorphism of STMS marker. Allelic polymorphism of STMS markers among multiple wheat (A-H) and rice (I, J) genotypes on agarose gels: A) Xgwm 282–7A, B) Xgwm 162–3A, C) Xgwm 304–5A, D) Xgwm 169–6A, E) Xgwm 99–1A, F) Xgwm 136–1A, G) Xgwm 135–1A, H) Xgwm 155–3A, I) RM 259, and J) RM 447. Numbers on the top indicates the wheat (A-H) and rice (I, J) genotypes as mentioned in the Table 1. Lane ‘M’ indicates 100 bp DNA ladder.

The SYBR Green melt-profiling approach was further evaluated for differentiation between alleles of STMS markers in both wheat and rice, using multiple genotypes (Table 1). Several STMS amplicons exhibited length polymorphism among the wheat (Fig 3A–3H, Table 2) and rice genotypes (Fig 3I and 3J, Table 2).

**Fig 4:**
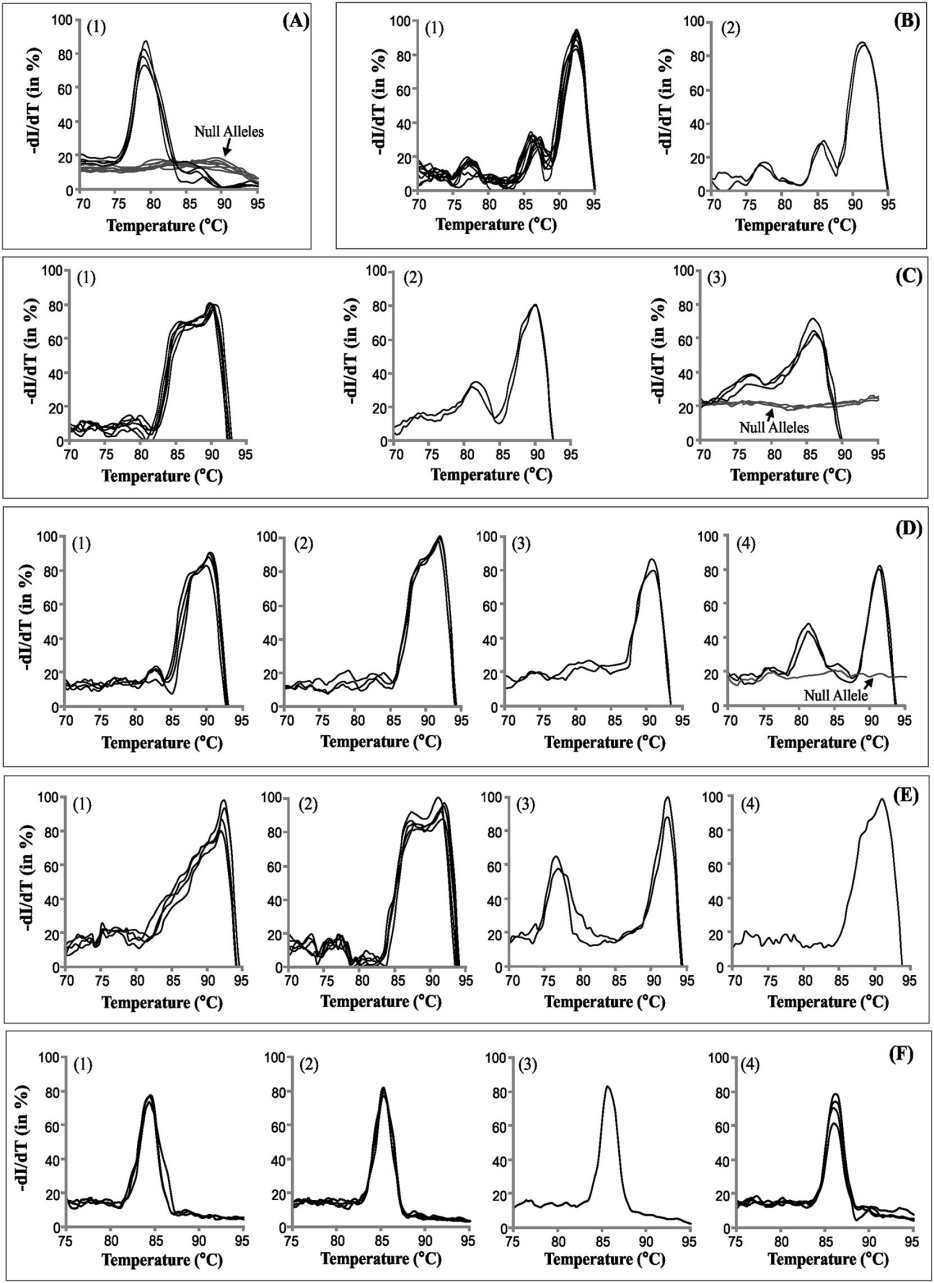
STMS allele differentiation by SYBR Green melt-profiling. **S**YBR Green melt-profiles of alleles of STMS markers among twelve wheat (A-E) and rice (F) genotypes: A) wheat STMS Xgwm 162–3A, B) wheat STMS Xgwm 304–5A, C) wheat STMS Xgwm 169–6A, D) wheat STMS Xgwm 282–7A, E) wheat STMS Xgwm 448–2A, and F) rice STMS RM 447. Multiple melt-profiles of STMS marker alleles (indicative of variation in Tm and curve shape difference) are indicated by number (1–4) while null alleles (if any) are indicated by arrows.

In addition, both wheat and rice STMS showed few cases of monomorphic alleles (Xgwm 135–1A, Xgwm 357–1A) or null alleles (Xgwm 162–3A, Xgwm 169–6A, Xgwm 264–1B, Xgwm 368–4B) (Fig 3, S3 Fig, Table 2). Alleles of sixteen wheat STMS and seven rice STMS markers were subjected to SYBR Green melt-profiling to assess its utility in differentiating them. The melt-profiles were compared for variation in Tm, profile shape or both. Among the twelve wheat genotypes Xgwm 162–3A, showed single type allele or null allele (Fig 4A), while Xgwm 304–5A showed two distinct allelic profiles with minor difference in Tm (Fig 4B). Similarly, STMS maker Xgwm 169–6A showed three profiles and a null allele, (Fig 4C), Xgwm 282–7A showed four profiles and a null allele (Fig 4D), and Xgwm 448–2A showed four profiles with variable features (Fig 4E). Multiple allele specific melt-profiles were also obtained for of STMS RM 447 among twelve rice genotypes (Fig 4F).

**Fig 5:**
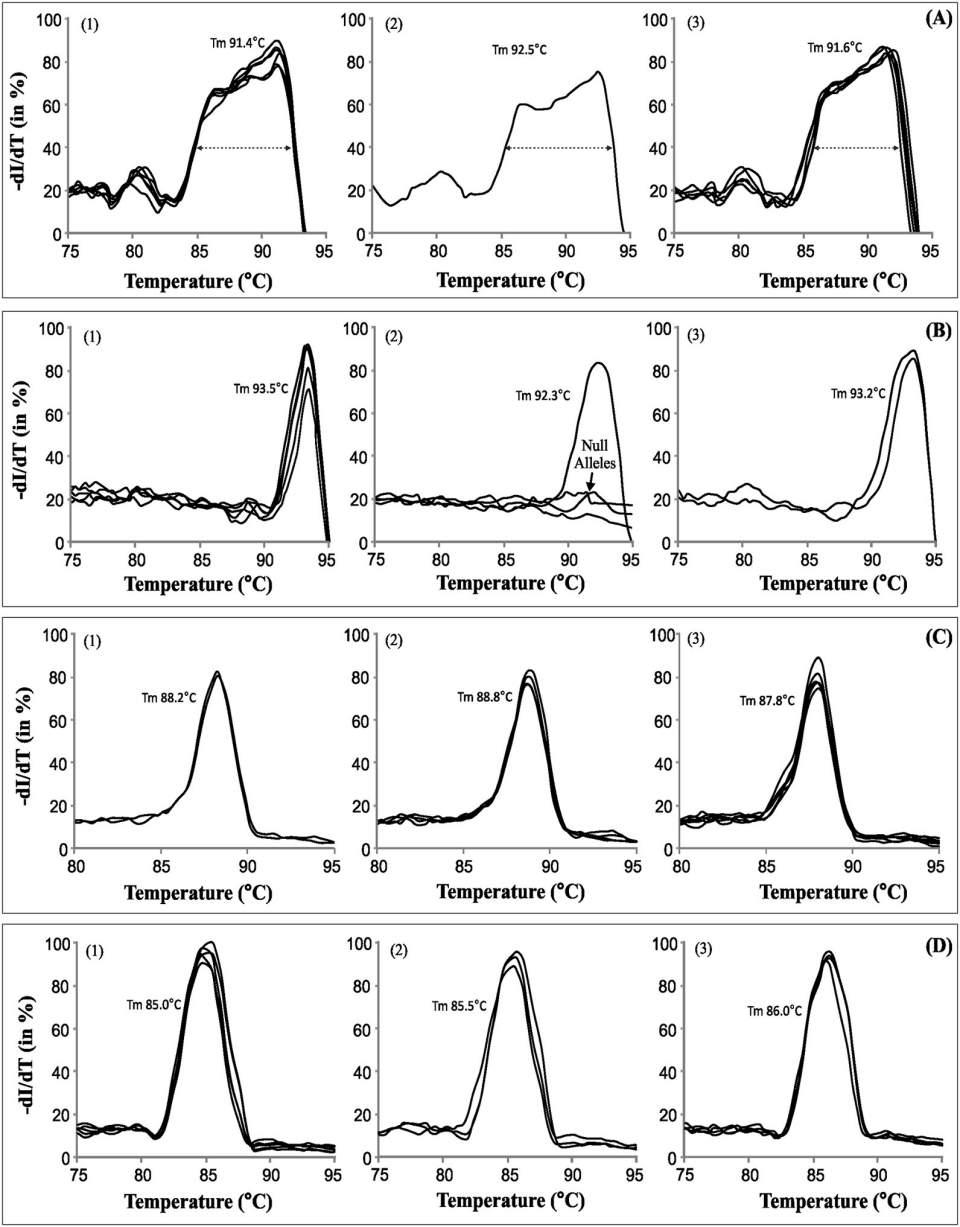
SYBR Green melt-profiles of STMS with low length polymorphism. SYBR Green melt-profiles of STMS marker alleles that showed low/no length polymorphism among twelve wheat genotypes: A) Xgwm 357–1A and B) Xgwm 232–1D. SYBR Green melt-profiles of STMS marker alleles that showed low/no length polymorphism among twelve rice genotypes: C) RM 154 and D) RM 431. Numbers indicate the profiles obtained for each STMS along with the corresponding Tm values.

Allelic differentiation capability of SYBR Green melt-profiling was evaluated for six STMS markers that show low/no length polymorphism (allelic homoplasy) on agarose gel (S3 Fig). The SYBR Green melt-profiles could differentiate (on basis of Tm or curve shape or both) STMS alleles of certain markers with low or no allelic variability on gels, in wheat (Fig 5A and 5B) and rice (Fig 5C and 5D). Collectively, these results show the feasibility of differentiation of STMS alleles by melt-profiling, where a null allele can be differentiated in a straightforward manner, while others can be differentiated on the basis of Tm, melt-profile characteristics or both.

### SYBR Green melt-profiling based multiplexing of STMS markers

**Fig 6:**
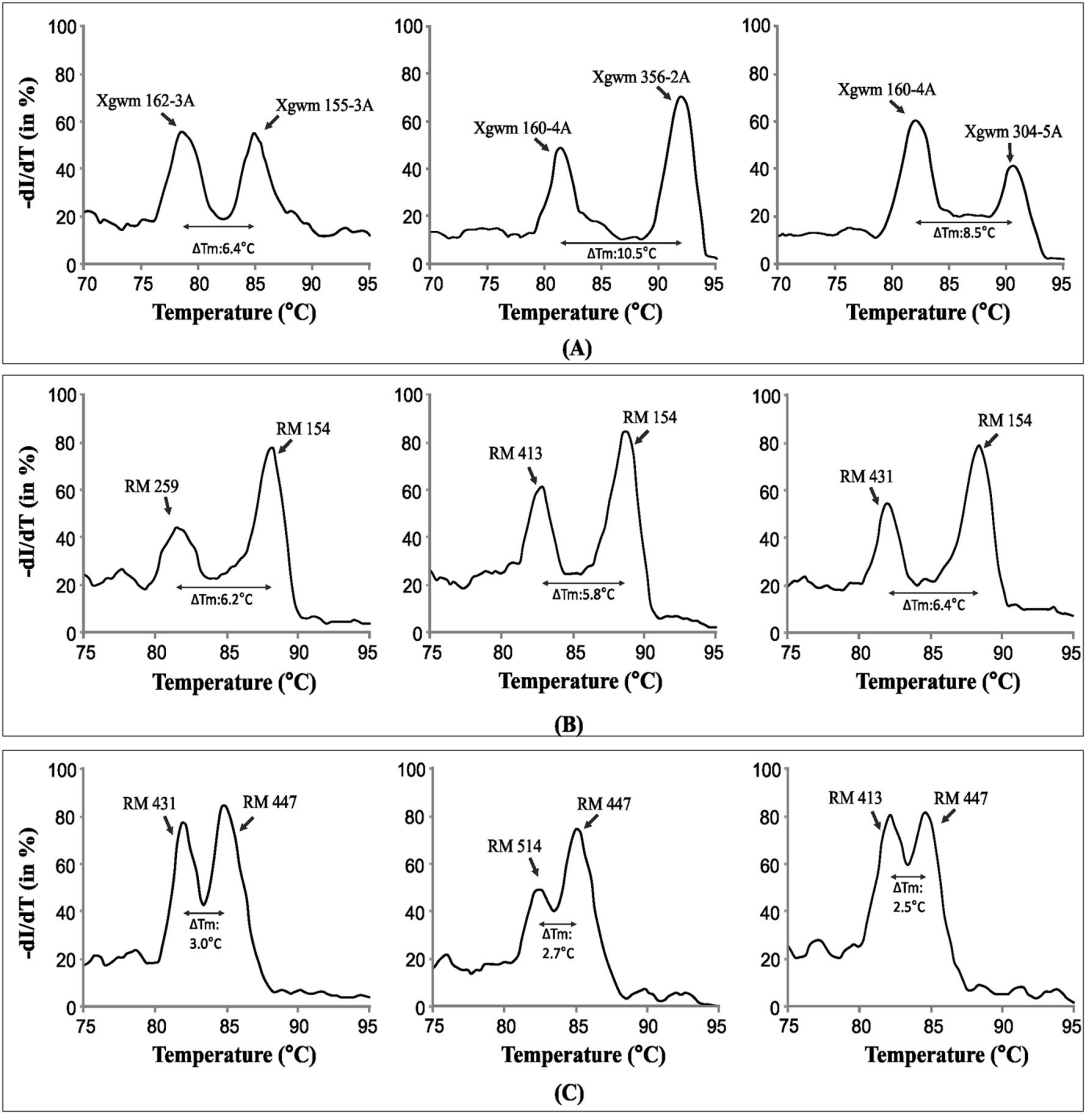
Multiplex STMS analysis. SYBR Green melt-profiles generated in multiplex PCR of STMS marker pairs in wheat and rice: A) multiplex analysis of three pair of wheat STMS markers with ΔTm > 5 °C, B) multiplex analysis of three pair of rice STMS markers with ΔTm > 5 °C, C) multiplex analysis of three pair of rice STMS markers with ΔTm < 5 °C. ΔTm is ‘melting temperature difference’. Designation of STMS used in multiplex assays are shown in each melt-profile plot and arrows indicate the STMS specific peaks.

Feasibility of SYBR Green melt-profiling for multiplex analysis was evaluated for STMS marker pairs that showed non-overlapping melt-profiles with melting temperature difference (ΔTm) of > 5 °C. Several such wheat STMS marker pairs were optimized for multiplex PCR (S4 Fig). The multiplexed STMS products when subjected to SYBR Green melt-profiling yielded distinct signal peaks specific to individual STMS that were included in the multiplex assay. SYBR Green multiplex melt-profiles of three pairs of wheat STMS markers with ΔTm > 5 °C (Xgwm 162–3A+Xgwm 155–3A, Xgwm 160–4A+Xgwm 356–2A, Xgwm 160–4A+Xgwm 304–5A) are shown in Fig 6A. Multiplex analysis was also carried out for six rice STMS pairs, of which three with ΔTm > 5 °C (RM 259+RM 154, RM 413+RM 154, RM4 31+RM 154) are shown in Fig 6B. Results showed that STMS marker pairs with ΔTm of ≥ 5 °C can yield well resolved peaks. Furthermore, STMS marker pairs (RM 431+RM 447, RM 447+RM 514, RM 447+ RM 413) with ΔTm value < 5 °C also showed STMS marker specific peaks but not completely resolved (Fig 6C). This demonstrates the feasibility of SYBR Green melt-profiling for STMS multiplex assay, thereby extending the utility of GLADS approach. However, multiplexing was not feasible for markers with overlapping melt-profiles or low ΔTm value.

## Discussion

This study shows the utility of SYBR Green dye based melt-profiling as a simple, convenient and low-cost gel-less approach for detection of STMS markers (referred to as GLADS). Crop-breeding involves multiple experiments (executed in parallel and involve large populations) that are routinely carried out using laborious and time-consuming conventional methodologies. DNA maker assisted selection (MAS) allow rapid screening of linked desirable traits and reduce overall breeding time [17], Among the PCR markers, STMS are preferable in breeding experiments due to their co-dominant nature, high abundance, wide genome coverage, and transferability [14–15,18]. At present, most STMS studies primarily rely on low-throughput, laborious, and time-consuming agarose or polyacrylamide gels [40–43]. This is an important concern while handling large crop populations [17,44], thereby limiting the full potential of the STMS based MAS.

Several gel-free STMS detection methods have been reported, however many of these are either technically intensive or rely on specialized consumables/hardware/software that makes these expensive for large scale analysis as in crop breeding (S2 Table). The marker screening approach need to be technically simple, convenient, breeder friendly and cost effective for analysis of large experimental populations [4,45]. SYBR Green based GLADS approach reported here addresses such these concerns for easy STMS analysis. Many previously reported STMS detection methods are often technically intensive and economically not viable for large scale usage. For example, STMS analysis using fluorescently labeled primers automate the scoring but high cost of labeling, expensive detection systems comprise major negative factors [28,29]. Similarly, capillary electrophoresis for automated and high-throughput analysis [27] also need specialized instrumentation and consumables resulting in high running cost (S2 Table). Sensitive approaches like TaqMan assays, molecular beacons, scorpions probes are more appropriate for small scale studies, and some of these require primers to be redesigned for effective detection [6,46–48]. Similarly, utility of HRM has also been shown for diverse applications including STMS analysis [12,32,49–53]. it uses expensive dyes, may require primer resigning, and rely on specific real-time hardware/software that might have limited its widespread usage [34]. The present GLADS approach, on the other hand simplifies the detection using a convenient, user friendly and relatively simple setup using already available primers.

For two major aspects of DNA marker work (development of new and use of available markers), convenience and cost-effectiveness of an approach ensure its easy adoption and widespread use. For example, in several MAS experiments, tightly linked STMS (to a trait) are often ascertained on gel-based methods for several generations [38,54–57]. In such cases, the GLADS approach offer a simple, convenient gel-less STMS detection alternative. It can be effective in utilization of available STMS markers linked to important traits in wheat (http://maswheat.ucdavis.edu/protocols/index.htm), rice (http://www.ricebase.org/ http://www.gramene.org) and other crops. This SYBR Green based approach ascertains the presence/absence of STMS and differentiates STMS markers/alleles solely by melting-profiles, in a gel-free manner.

DNA binding dyes (ethidium bromide, SYBR Green, LC Green etc.) can be utilized for STMS detection in a post-PCR end-point assay. Hernández and coworkers demonstrated in-tube detection of STMS by addition of ethidium bromide directly in the PCR tubes [31]. However, the end-pint assays cannot differentiate the specific signal (due to an amplicon) from false non-specific signal (due to primer-dimers, unused primers and template). Additionally, ethidium bromide has lower sensitivity, inhibits PCR and is carcinogenic in nature. The present two-step approach first optimized the use of SYBR Green (more sensitive and safe dye than ethidium bromide) in the PCR step, followed by a melt-profiling step to ascertain the signal specificity. The melt-profiling allows differentiation of amplicon specific and non-specific signal [19,46,58], which is not feasible in an end-point method [24]. The SYBR Green based GLADS is flexible in execution: the dye can be included in the PCR assay mix and the samples can be subjected to post-PCR melt-profiling, with complete analysis on a real-time PCR or alternatively the PCR can be performed on a regular machine followed by addition of dye and melt-profiling on a real-time PCR instrument.

Real time PCR assays for gene expression analysis carry out melt-profiling to assess amplicon specificity [34,59,60], while GLADS uses it for STMS analysis. Unlike a gel that analyzes amplicons for length polymorphism, the melt-profiles reflect variation due to a combination of length, %GC, secondary structures [61], and generate marker-specific profiles useful for gel-less analysis. It is a sensitive and useful assay but needs to be optimized to minimize non-specific interference due to certain parameters. The non-specific binding of SYBR Green is affected by salt concentration, and DNA composition [61,62]. It may also show inhibitory effect on PCR [63]. Optimized melt-profiling assay show lower problems due to non-specific peaks and spikes [59], as also seen in the present study. The SYBR Green melt-profiling for STMS detection was also helpful for allele differentiation, which is important in cases where a particular allele is linked to a desirable trait [11,12]. The assay differentiated between a STMS allele (signal peak) and a null allele (no signal peak) in a straightforward manner and showed capability to detect polymorphism among STMS alleles. However, discrimination was not feasible between alleles with overlapping melt curves.

Present approach is cost-effective and saves time as evident from the comparison of several approaches for STMS analysis (S2 Table). For example, analysis of 96–384 samples (on a Roche LC 480 II or other high-throughput systems) can be completed in 30 minutes which is considerably faster than traditional gel based methods. It is economical (20–51% lower cost) than several other gel and non-gel based methods (S2 Table). For analysis of large number of markers/experimental samples cost of analysis is a critical factor. The economy of the SYBR Green melt-profiling is enhanced by multiplexing that further reduces the cost, time and efforts [present study, 19,56]. Multiplex PCR often require re-designed primers, however in the present study it was successfully carried out for STMS marker pairs showing non-overlapping melt-profiles (ΔTm > 3.0 °C), using existing primer combinations.

Due to simple and low cost, the SYBR Green melt-profiling assays have been utilized in various studies viz. pathogen identification [64], analysis of ISBP (insertion site-based polymorphism) markers [36], SCAR marker detection [19], and SNP marker analysis [65,66]. Present study further extends the scope of simple SYBR Green melt-profiling approach for gel-less detection of STMS markers. The usage of real-time PCR based marker analysis is limited to small numbber of crop breeding studies viz. hybridization probes based analysis [67], oleic acid content linked maker analysis [8], HRM analysis [68,69], primarily due to high cost associated with the consumables used. Simplified approaches like SYBR Green based GLADS can enhance the usage of real-time PCR based analysis in crop breeding experiments for markers like STMS (this study) and SCARs [19], which is otherwise mostly restricted to analysis of gene expression [34] and ascertaining transgene copy number [70].

Overall, use of GLADS for STMS analysis is more advantageous than cumbersome long-format polyacrylamide or high-resolution agarose gels. It is more convenient, economical and breeder friendly than alternative technologically intensive or expensive methods for crop breeding applications. GLADS can be used as a stand lone approach or it can be a part of an integrated STMS analysis scheme. It can be employed initially to assess the STMS suitable for melt-profiling analysis. Such markers can be directly analyzed by GLADS approach, while remaining can be scored by alternative approaches or a combination of methods. The approach will be useful in specialized applications such as, MAS (use single trait-linked STMS marker) and MABB (involve scoring of several STMS markers for background and foreground selection), in wheat, rice and other crops.

## Conclusions

The GLADS approach combined the simplicity of PCR, sensitivity of SYBR Green, and informativeness of melt-profiles for a simple, rapid and effective assay for various types of STMS analysis viz. detection/scoring, differentiation, allelic polymorphism, and multiplex analysis. It is relatively economical (20–51% lower cost than other approaches) and can be performed on a routine real-time PCR and without any specialized consumables/hardware/software requirements. The efficacy of GLADS can be assessed during initial phase of STMS analysis, and it can be integrated with existing methods. GLADS based STMS analysis seems a promising approach to reduce the dependence on cumbersome gel-based detection approaches in wheat, rice as well as other crops.

## Supporting information

S1 Fig Gradient PCR assay. Optimization of PCR amplification of some wheat STMS markers by gradient PCR: A) Xgwm 337–1D, B) Xgwm 136–1A, C) Xgwm 261–2D, D) Xgwm 264–1B, E) Xgwm 182–5D, F) Xgwm 33–1B, G) Xgwm 190–5D, H) Xgwm 114–3B, I) Xgwm 356–2A, J) Xgwm 174–5D. The PCR amplified products were analysed on agarose gel. The numbers on the top of the lanes indicate the annealing temperature used in the gradient PCR.
(TIF)

S2 Fig Effect of annealing temperature on SYBR Green melt-profiles. SYBR Green melt-profiles of three wheat STMS markers subjected to gradient PCR analysis: A) Xgwm 155–3A, B) Xgwm 369–3A, C) Xgwm 512–2A. Gradient PCR was carried from 56°C to 62°C as indicated by different coloured profiles. The first two markers (A, B) showed enhanced specificity at higher annealing temperature, while the third marker (C) showed a doublet profile at all temperatures.
(TIF)

S3 Fig Analysis of STMS alleles on agarose gel. Agarose gel profiles of PCR amplified STMS markers that did not show well resolved alleles. Top panel: Wheat STMS markers among 12 genotypes, A) Xgwm 357–1A, B) Xgwm 135–1A, C) Xgwm 337–1D, D) Xgwm 232–1D. Bottom panel: Rice STMS markers among 12 genotypes, E) RM 431, F) RM 154. Lane M: 100 bp DNA ladder.
(TIF)

S4 Fig STMS multiplex analysis. Analysis of some wheat STMS markers amplified by multiplex PCR assay on agarose gel: lane 1: Xgwm 459–6A+Xgwm 369–3A, lane 2: Xgwm 160–4A+Xgwm 356–2A, lane 3: Xgwm 160–4A+Xgwm 304–5A, lane 4: Xgwm 261–2D+Xgwm 232–1D, lane 5: Xgwm 190–5D+Xgwm 232–1D, lane 6: Xgwm 102–2D+Xgwm 111–7D, lane 7: Xgwm 261–2D+Xgwm 117–7D, lane 8: Xgwm 190–5D+Xgwm 117–7D, lane 9: Xgwm 261–2D+ Xgwm 608–4D, lane M: 100 bp DNA ladder. Arrows indicate the position of two STMS markers in each lane.
(TIF)

S1 Table List of STMS markers specific to wheat (A, B, and D-genome) and rice analyzed in the present study. (DOCX)

S2 Table Comparison of approximate cost (in USD), time of analysis, and other important aspects of SYBR Green based approach and few other techniques for STMS marker detection. (DOCX)

## Abbreviations

dNTP: Deoxynucleotide triphosphate
EST: Expressed Sequence Tag
MAS: Marker Assisted Selection
PAGE: Polyacrylamide Gel Electrophoresis
PCR: Polymerase Chain Reaction
QTLs: Quantitative Trait Loci
SSR: Simple Sequence Repeat
STMS: Sequence Tagged Microsatellite site
TBE: Tris-Borate EDTA
Tm: Melting Temperature
