# Supplementary figures and images for: GLADS: A gel-less approach for detection of STMS markers in wheat and rice
Source: PLoS One. 2019 Nov 5;14(11):e0224572. doi: 10.1371/journal.pone.0224572 (PMC6830750; doi:10.1371/journal.pone.0224572)

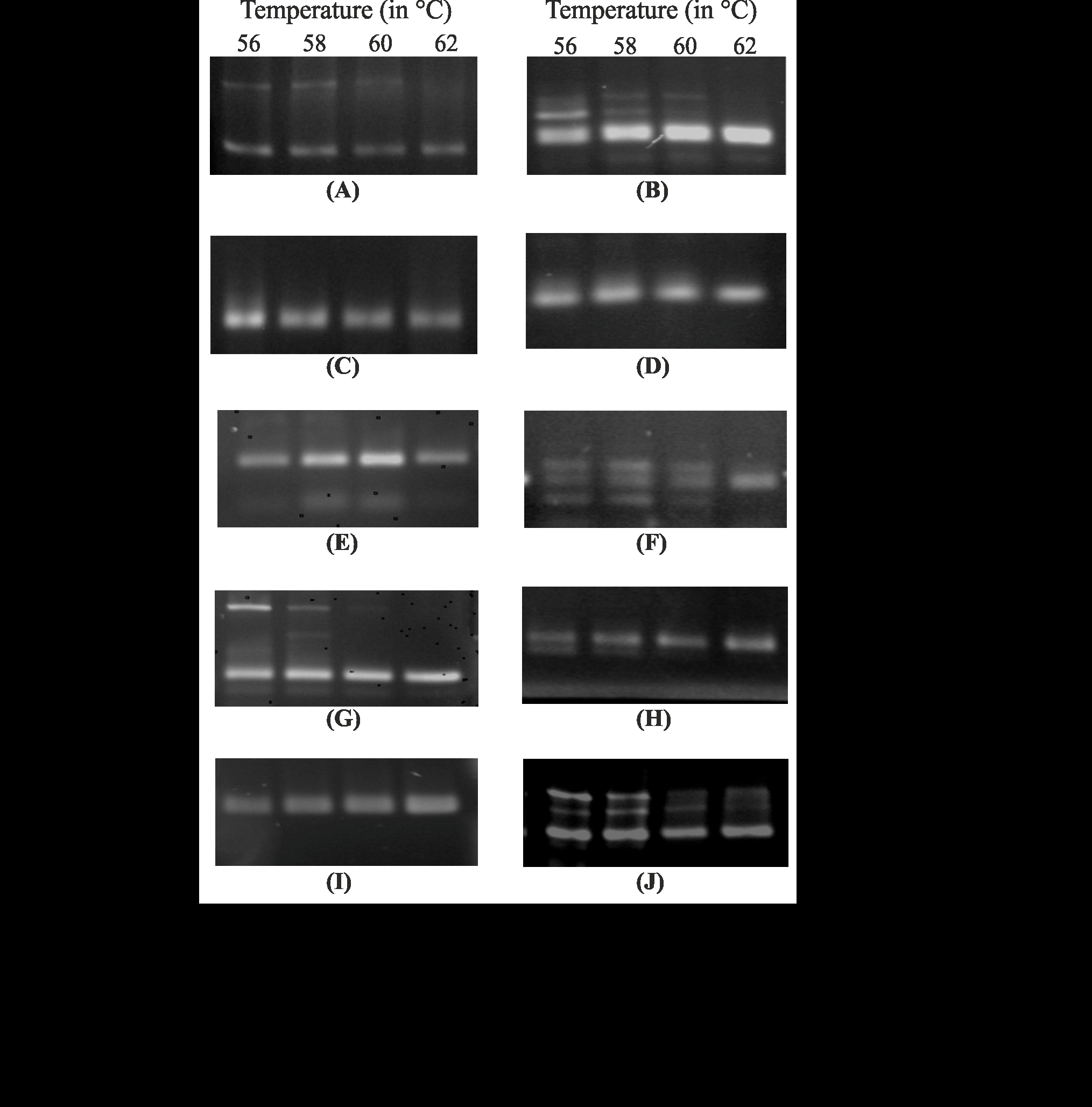

Supplement: S1 Fig — Optimization of PCR amplification of some wheat STMS markers by gradient PCR: A) Xgwm 337–1D, B) Xgwm 136–1A, C) Xgwm 261–2D, D) Xgwm 264–1B, E) Xgwm 182–5D, F) Xgwm 33–1B, G) Xgwm 190–5D, H) Xgwm 114–3B, I) Xgwm 356–2A, J) Xgwm 174–5D. The PCR amplified products were analysed on agarose gel. The numbers on the top of the lanes indicate the annealing temperature used in the gradient PCR. (TIF) [file pone.0224572.s001.tif]

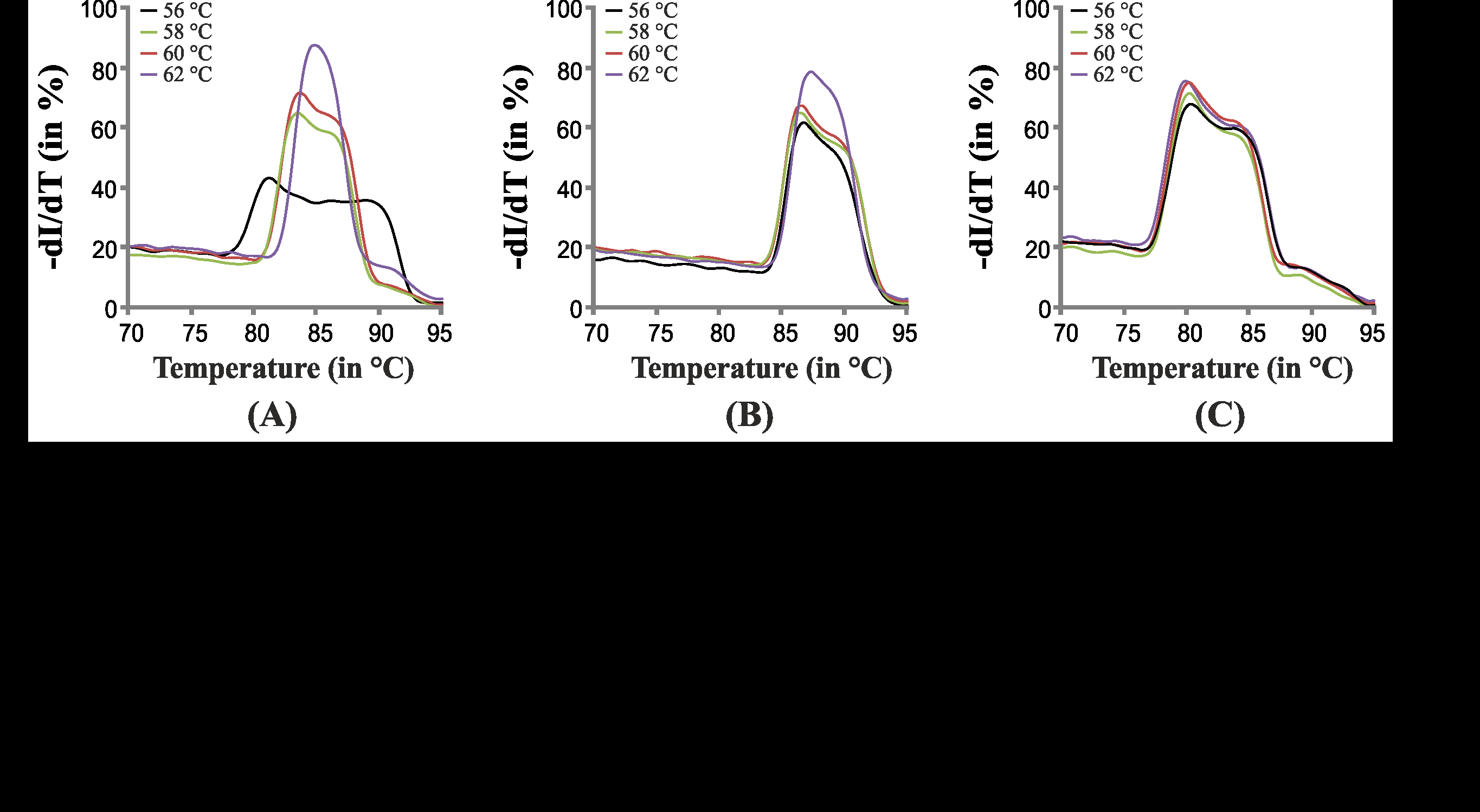

Supplement: S2 Fig — SYBR Green melt-profiles of three wheat STMS markers subjected to gradient PCR analysis: A) Xgwm 155–3A, B) Xgwm 369–3A, C) Xgwm 512–2A. Gradient PCR was carried from 56°C to 62°C as indicated by different coloured profiles. The first two markers (A, B) showed enhanced specificity at higher annealing temperature, while the third marker (C) showed a doublet profile at all temperatures. (TIF) [file pone.0224572.s002.tif]

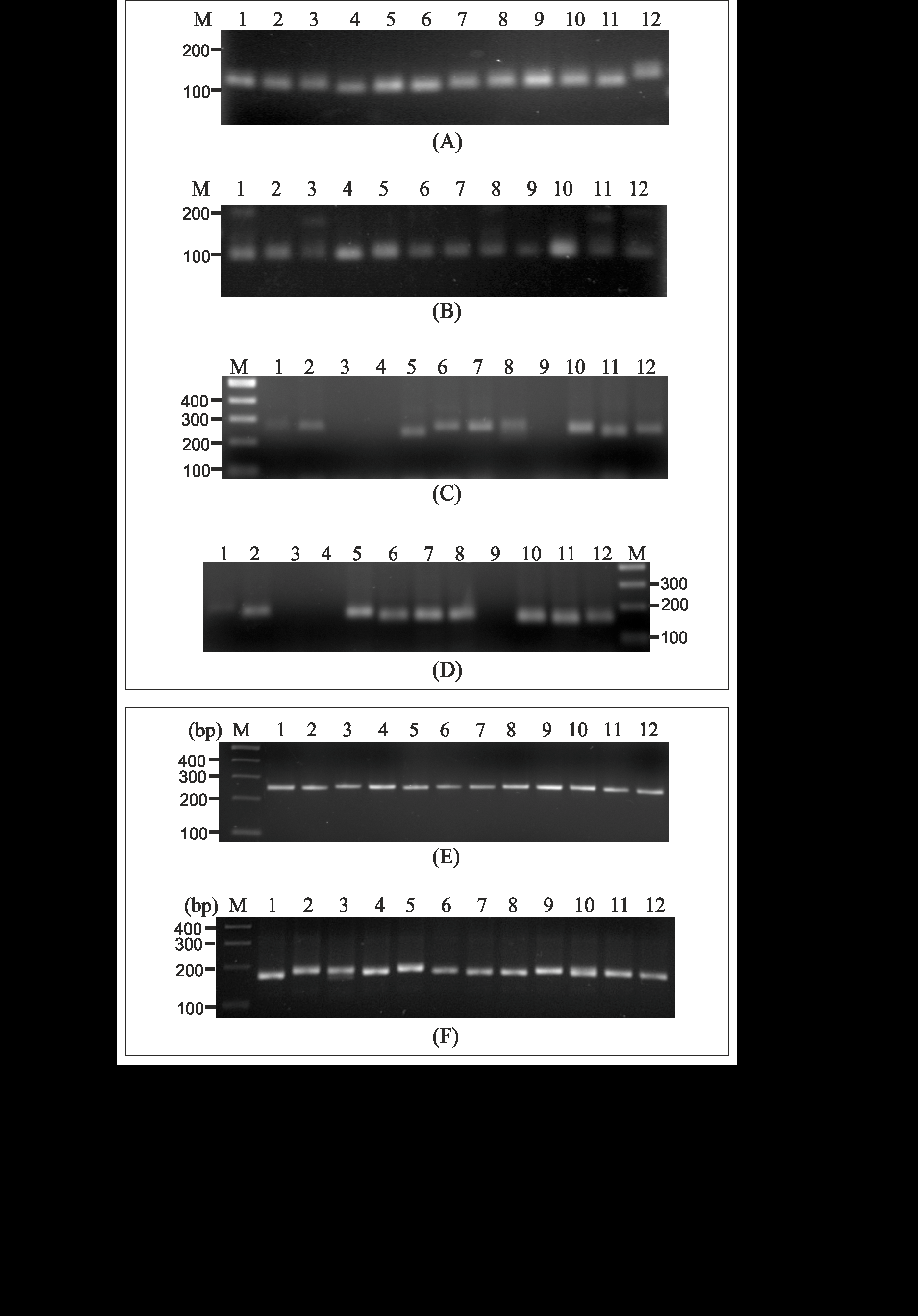

Supplement: S3 Fig — Agarose gel profiles of PCR amplified STMS markers that did not show well resolved alleles. Top panel: Wheat STMS markers among 12 genotypes, A) Xgwm 357–1A, B) Xgwm 135–1A, C) Xgwm 337–1D, D) Xgwm 232–1D. Bottom panel: Rice STMS markers among 12 genotypes, E) RM 431, F) RM 154. Lane M: 100 bp DNA ladder. (TIF) [file pone.0224572.s003.tif]

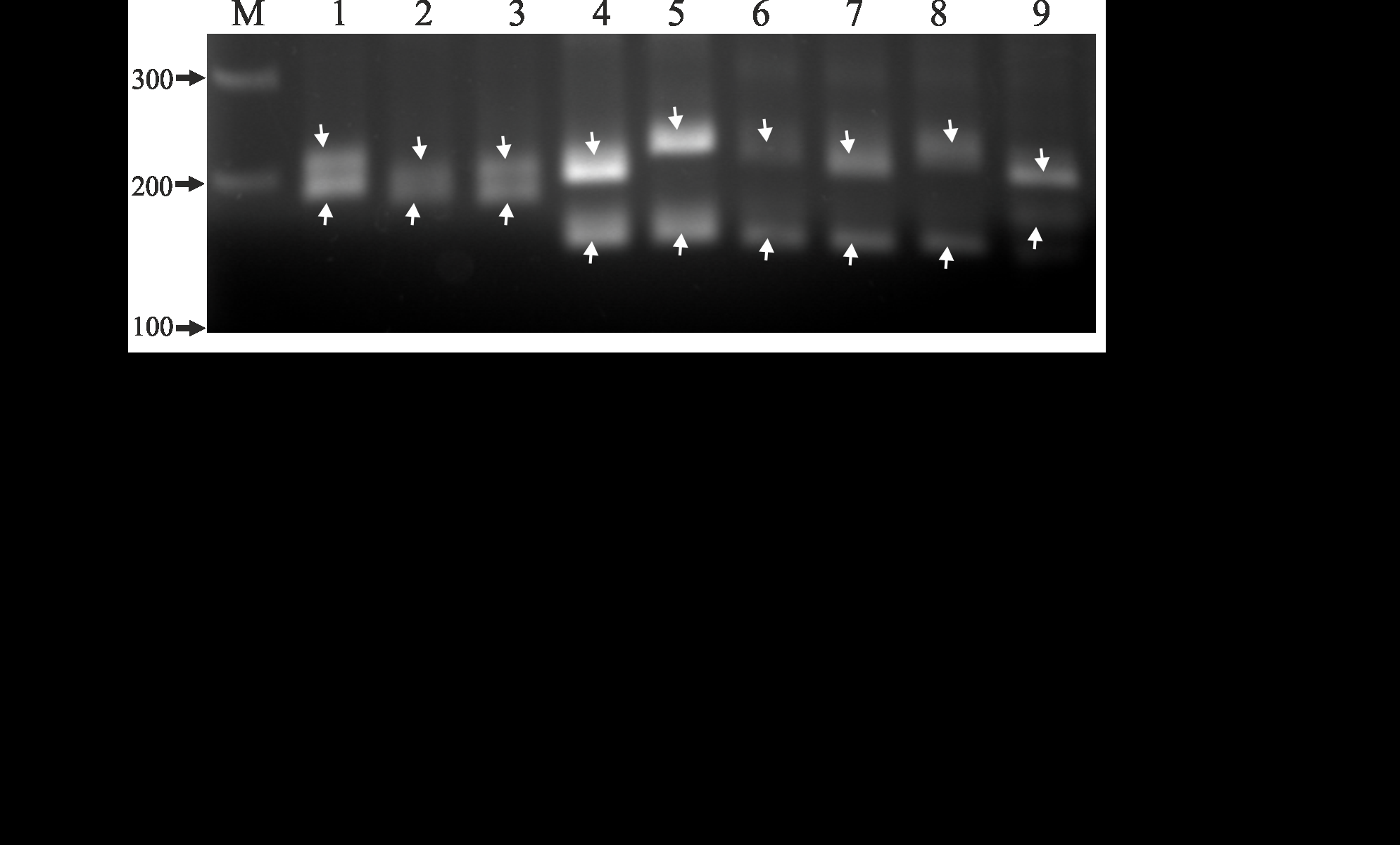

Supplement: S4 Fig — Analysis of some wheat STMS markers amplified by multiplex PCR assay on agarose gel: lane 1: Xgwm 459–6A+Xgwm 369–3A, lane 2: Xgwm 160–4A+Xgwm 356–2A, lane 3: Xgwm 160–4A+Xgwm 304–5A, lane 4: Xgwm 261–2D+Xgwm 232–1D, lane 5: Xgwm 190–5D+Xgwm 232–1D, lane 6: Xgwm 102–2D+Xgwm 111–7D, lane 7: Xgwm 261–2D+Xgwm 117–7D, lane 8: Xgwm 190–5D+Xgwm 117–7D, lane 9: Xgwm 261–2D+ Xgwm 608–4D, lane M: 100 bp DNA ladder. Arrows indicate the position of two STMS markers in each lane. (TIF) [file pone.0224572.s004.tif]
