# Supplementary material for: GLADS: A gel-less approach for detection of STMS markers in wheat and rice
Source: PLoS One. 2019 Nov 5;14(11):e0224572. doi: 10.1371/journal.pone.0224572 (PMC6830750; doi:10.1371/journal.pone.0224572)
Supplement: S1 Table — (DOCX) [file pone.0224572.s005.docx]

### S1 Table: List of STMS markers specific to wheat (A, B, and D-genome) and rice analyzed in the present study.

| S. No. | STMS marker | Species/  Genome | Characteristic |
| --- | --- | --- | --- |
| 1 | Xgwm 136-1A | Wheat A-Genome | (CT)58 |
| 2 | Xgwm 357-1A |  | (GA)18 |
| 3 | Xgwm 135-1A |  | (GA)20 |
| 4 | Xgwm 99-1A |  | (CA)21 |
| 5 | Xgwm 265-2A |  | (GT)23 |
| 6 | Xgwm 448-2A |  | (GA)29 |
| 7 | Xgwm 155-3A |  | (CT)19 |
| 8 | Xgwm 162-3A |  | (CA)14AA(CA)4 |
| 9 | Xgwm 160-4A |  | (GA)21 |
| 10 | Xgwm 156-5A |  | (GT)14 |
| 11 | Xgwm 304-5A |  | (CT)22 |
| 12 | Xgwm 459-6A |  | (GA)>28 |
| 13 | Xgwm 570-6A |  | (CT)14(GT)18 |
| 14 | Xgwm 169-6A |  | (GA)23 |
| 15 | Xgwm 282-7A |  | (GA)38 |
| 16 | Xgwm 356-2A |  | (GA)36 |
| 17 | Xgwm 369-3A |  | (CT)11(T)2(CT)21 |
| 18 | Xgwm 397-4A |  | CT21 |
| 19 | Xgwm 5-3A |  | (TC)23(T)4(GT)12(GA)10 |
| 20 | Xgwm 512-2A |  | (GT)16 |
| 21 | Xgwm 140-1B | Wheat B-Genome | (CT)42 |
| 22 | Xgwm 264-1B |  | (CA)9A(CA)24 |
| 23 | Xgwm 257-2B |  | (GT)30 |
| 24 | Xgwm 114-3B |  | (GA)53 |
| 25 | Xgwm 547-3B |  | (CA)12 |
| 26 | Xgwm538-4B |  | (GT)6(T)(GT)10 |
| 27 | Xgwm 368-4B |  | (AT)25 |
| 28 | Xgwm 68-5B |  | (GA)3(G)3(GA)25 |
| 29 | Xgwm 251-4B |  | (CA)28 |
| 30 | Xgwm 219-6B |  | (GA)35imp |
| 31 | Xgwm 66-4B |  | (CA)30(TA)21 |
| 32 | Xgwm 210-2B |  | (GA)20 |
| 33 | Xgwm 165-4B |  | (GA)20 |
| 34 | Xgwm 33-1B |  | (GA)19 |
| 35 | Xgwm 46-7B |  | (GA)2GC(GA)33 |
| 36 | Xgwm 337-1D |  | (CT)5(CACT)6(CA)43 |
| 37 | Xgwm 232-1D | Wheat D-Genome | (GA)19 |
| 38 | Xgwm 642-1D |  | (GT)14 |
| 39 | Xgwm 320-2D |  | (GT)9(GA)15 |
| 40 | Xgwm 645-3D |  | (CT)23imp |
| 41 | Xgwm 52-3D |  | (GT)4AT(GT)20 |
| 42 | Xgwm 608-4D |  | (GA)16 |
| 43 | Xgwm 182-5D |  | (CT)18 |
| 44 | Xgwm 174-5D |  | (CT)22 |
| 46 | Xgwm 192-5D |  | (CT)46 |
| 47 | Xgwm 190-5D |  | (CT)22 |
| 48 | Xgwm 55-6D |  | (TC)3(T)3(CT)17 |
| 49 | Xgwm 111-7D |  | (CT)32(GT)17 |
| 50 | Xgwm 469-6D |  | (CT)19(CA)10 |
| 51 | Xgwm 349-3D |  | (GA)34 |
| 52 | Xgwm 428-7D |  | (GA)22 |
| 53 | Xgwm 484-2D |  | (CT)29 |
| 54 | Xgwm 102-2D |  | (CT)15 |
| 55 | Xgwm 261-2D |  | (CT)21 |
| 56 | RM 55 |  | (GA)17 |
| 57 | RM 154 | Rice | (GA)21 |
| 58 | RM 431 |  | (AG)16 |
| 59 | RM 413 |  | (AG)11 |
| 60 | RM 3481 |  | (CT)22 |
| 61 | RM 133 |  | (CT)8 |
| 62 | RM 17467 |  | (AT)31 |
| 63 | RM 26063 |  | (CT)33 |
| 64 | RM 279 |  | (GA)16 |
| 65 | RM 20236 |  | (AG)10 |
| 66 | RM 585 |  | (ATA)29 |
| 67 | RM 228 |  | (CA)6(GA)36 |
| 68 | RM 3403 |  | (CT)17 |
| 69 | RM 29 |  | (GA)7 |
| 70 | RM 408 |  | (CT)13 |
| 71 | RM 514 |  | AC(12) |
| 73 | RM 259 |  | (CT)17 |
| 74 | RM 447 |  | (CTT)8 |

*Sizes of STMS amplicons were estimated by ‘GeneTools’ software (Syngene, UK), imp: imperfect repeat
