# Supplementary material for: GLADS: A gel-less approach for detection of STMS markers in wheat and rice
Source: PLoS One. 2019 Nov 5;14(11):e0224572. doi: 10.1371/journal.pone.0224572 (PMC6830750; doi:10.1371/journal.pone.0224572)
Supplement: S2 Table — (DOCX) [file pone.0224572.s006.docx]

**S2 Table** Comparison of approximate cost (in USD), time of analysis, and other important aspects of SYBR green based approach and few other techniques for STMS marker detection.

| Component of Assay & Analytical Cost of 100 samples | Gel and Gel-less Approaches for STMS Detection and Analysis | | | | |
| --- | --- | --- | --- | --- | --- |
|  | Agarose gel electrophoresis^2^ | Polyacrylamide gel^3^ electrophoresis (PAGE) | Capillary array electrophoresis (CAE) | High-resolution Melting (HRM) | SYBR Green melt-profiling^4^ |
| Cost of PCR^1^ | 89.40 | 89.40 | 89.40 | 112.11  (includes cost of PCR reagents & HRM dye) | 89.40 |
| Cost of fluorescent dye/ master mix reagent | Not applicable | Not applicable | Not applicable |  | 0.18 |
| Cost of electrophoresis and staining | 71.10 | 62.22 | Not applicable | Not applicable | Not applicable |
| Cost of capillary array cartridge | Not applicable | Not applicable | 94.70 | Not applicable | Not applicable |
| Total cost of analysis | 160.50 | 151.62 | 184.10 | 112.11 | 89.58 |
| Time required (in hours) | 6h  6h (96 samples) to 24h (384 samples) (~1.5h/gel) | 24h  24h (96 samples) to 96h (384 samples) (24h/gel) | 1h  1h (96 samples) to 4h (384 samples) (1h/96-well plate) | 0.5h  (96-384 samples) | 0.5h  (96-384 samples) |
| Hardware requirements | Agarose electrophoresis apparatus & Gel-documentation system | PAGE apparatus &  Gel-documentation system | Capillary array electrophoresis (CAE) system | HRM compatible real time PCR instrument | Regular real time PCR instrument |
| Software requirements | 1-D gel image analysis software | 1-D gel image analysis software | CAE data analysis software | HRM data analysis software | Regular real-time data analysis software |
| Technical skill requirements | Simple laboratory skills for setup and analysis | Advanced laboratory skills for handling large format PAGE gels | Advanced laboratory skills for handling CAE instrumentation | Simple laboratory skills | Simple laboratory skills |
| Advantages | Simple set up needed, convenient analysis, no primer redesigning | High-throughput than agarose; good resolution and cost-effective, no primer redesigning | High-throughput, convenient method, no dependence on gels, no primer redesigning | High-throughput, convenient and sensitive analysis, no dependence on gels | Simple, convenient, cost effective, high-throughput, no primer redesigning, no dependence on gels |
| Disadvantages | Low throughput, cumbersome with large sample size, not cost effective | Time-consuming, handling of large format PAGE gels difficult, cumbersome with large sample size | High cost of array consumables, may compromise on resolution  (< 3bp is difficult) | High cost of HRM consumables, specialized software, may require primer redesigning for amplicons | Simple melt-profiles, resolution may be less for certain markers |

^1^ Cost of PCR takes into accounts the price of all components used in PCR assay which is same for all approaches except HRM analysis (uses a specialized reaction mix); ^2^Cost of agarose gel electrophoresis estimation is based on price of high-resolution agarose (Sigma-Aldrich) in addition to other chemicals needed for running gels and staining; ^3^Cost of PAGE is inclusive of chemicals needed for plate preparation, gel-preparation and casting, running, fixing and staining; ^4^ Cost of SYBR Green for melt-profiling was estimated based on usage of dye (cat no: S9430, Sigma-Aldrich, 10,000X concentration) at a final concentration of 1X.
